# L-shaped association of dietary inflammatory index (DII) and chronic diarrhea: results from NHANES 2005–2010

**DOI:** 10.1186/s12889-025-21292-8

**Published:** 2025-01-08

**Authors:** Qing Zhao, Yue Xu, Xiangrui Li, Xiaotian Chen

**Affiliations:** Department of Clinic Nutrition, Nanjing Drum Tower Hospital, Affiliated Hospital of Nanjing University Medical School, Nanjing, Jiangsu China

**Keywords:** Chronic diarrhea, Dietary inflammatory index, NHANES, Cross-sectional study

## Abstract

**Background:**

Since diet is a known modulator of inflammation, the Dietary Inflammatory Index (DII), which quantifies the inflammatory potential of an individual’s diet, becomes a significant parameter to consider. Chronic diarrhea is commonly linked to inflammatory processes within the gut. Thus, this study aimed to explore the potential link between DII and chronic diarrhea.

**Methods:**

This research utilized data from the National Health and Nutrition Examination Survey (NHANES) 2005–2010. The DII was calculated according to the average intake of 28 nutrients using information gathered from two 24-hour recall interviews. The Bristol Stool Form Scale (BSFS) was adopted to describe chronic diarrhea, identifying stool Type 6 and Type 7. Multivariate logistic regression models examined the causal connection between DII and chronic diarrhea. Additionally, subgroup analyses and interaction tests were conducted.

**Results:**

The study encompassed 11,219 adults, among whom 7.45% reported chronic diarrhea. Initially, multivariate logistic regression analysis revealed a positive association between DII and chronic diarrhea. Nevertheless, this connection lost statistical significance (OR = 1.00; 95% CI, 0.96–1.05; *P* = 0.8501) after adjusting for all confounding variables. Stratified by sex, the analysis revealed a notable rise in the risk of chronic diarrhea with increasing DII among female participants (all *P* for trend < 0.05). This tendency remained constant even after full adjustment (*P* for trend = 0.0192), whereas no significant association was noted in males (all *P* for trend > 0.05). Furthermore, an L-shaped association emerged between DII and chronic diarrhea, with an inflection point of -1.34. In the population with DII scores below -1.34, each unit increase in DII correlated with a 27% reduction in the probability of chronic diarrhea (OR = 0.73; 95% CI, 0.57–0.93), whereas in the population with DII scores above -1.34, the risk increased by 4% (OR = 1.04; 95% CI, 0.98–1.10). Merely, the gender interaction was shown to be statistically significant based on subgroup analyses and interaction tests.

**Conclusions:**

A favorable association between DII and chronic diarrhea exists in adults in the United States. Nevertheless, additional long-term prospective studies are required to confirm and solidify those findings.

## Introduction

Chronic diarrhea affects up to 5% of the world’s population [1]. It can be defined by stools’ frequency, thinness, volume, or weight. However, quantifying this in clinical settings poses challenges. Typically, clinicians rely on tools like the Bristol Stool Form Scale (BSFS) to evaluate chronic diarrhea [2]. Chronic diarrhea is the primary symptom of both irritable bowel syndrome (IBS) [3] and inflammatory bowel disease (IBD) [4]. Distinguishing between patients with chronic diarrhea hinges on identifying whether the cause is functional or organic. In addition, certain dietary components can trigger or exacerbate chronic diarrhea [2]. Individuals with diarrhea often tend to consume more unhealthy plant-based foods like fruit juices and refined grains, leading to a reduction in gut microbiota diversity and a slight increase in pro-inflammatory bacterial strains [5]. Dietary guidelines recommend adopting regular meal patterns, limiting high-fiber food intake, and reducing alcohol, caffeine, and carbonated beverage consumption to alleviate IBS symptoms in about half of patients [6]. Thus, obtaining a detailed dietary history from patients is imperative.

The emergence of the Dietary Inflammatory Index (DII) offers a quantitative method for studying the link between chronic diarrhea and inflammatory diets. This index evaluates how food components impact inflammatory markers [7], categorizing diets as either pro-inflammatory or anti-inflammatory. Utilizing the DII provides a more comprehensive assessment of the inflammatory potential of one’s diet, given that a daily diet consists of complex food combinations rather than individual nutrients and foods. DII scores are typically computed using food frequency questionnaires (FFQ) [8], and a higher score indicates a more substantial inflammatory potential of dietary components [7]. Extensive research has examined the DII in various diseases. Increased DII has been positively correlated with cancer risk and death [9–12]. For instance, in colorectal cancer patients, each 1-point increase in DII score is associated with a 1.34-fold increase in colorectal cancer risk [13].

Moreover, a large prospective cohort study in the United States found that individuals with the highest tertile DII score had a 46% higher likelihood of dying from cardiovascular disease and higher all-cause mortality [14]. The DII also exhibits strong associations with other diseases such as type 2 diabetes [15], obesity [16], depression [17], and chronic obstructive pulmonary disease [18]. Recent studies have begun to unravel the intricate relationship between diet-induced inflammation and various gastrointestinal disorders. The findings of Salari-Moghaddam et al. demonstrated that adherence to a pro-inflammatory diet was associated with an increased risk of IBS [19]. Diet quality, as measured by the Adaptive Dietary Inflammation Index (ADII), was lower in patients with IBD and IBS, suggesting a common dietary link in the pathophysiology of these two diseases [20]. Furthermore, the results of a cross-sectional study demonstrated that a higher DII was significantly associated with constipation, with a positive and dose-related association [21]. The energy-adjusted dietary inflammatory index (E-DII), which accounts for energy intake, also showed a positive relationship with constipation [22]. Despite the significant impact of diet on gut health, the relationship between DII and chronic diarrhea remains unclear.

Given that chronic diarrhea significantly affects the quality of life and may reflect underlying inflammatory processes, understanding the potential impact of an inflammatory diet is critical. Focusing on National Health and Nutrition Examination Survey (NHANES) participants, this study aims to explore the relationship between chronic diarrhea and DII to understand better how pro-inflammatory dietary patterns may contribute to chronic diarrhea.

## Materials and methods

### Data source and study population

Data were gathered from NHANES, a population-based, nationwide cross-sectional survey meticulously crafted to scrutinize nutrition and health status in the United States, overseen by the National Center for Health Statistics (NCHS) [23]. To ensure the representativeness of the samples, a sophisticated multistage stratified probability sampling methodology on a biennial cycle was used. All NHANES research protocols were approved by the NCHS Research Ethics Review Board, and survey respondents (or, in the case of those under 16, their parents and legal guardians) provided written informed permission.

**Fig. 1:**
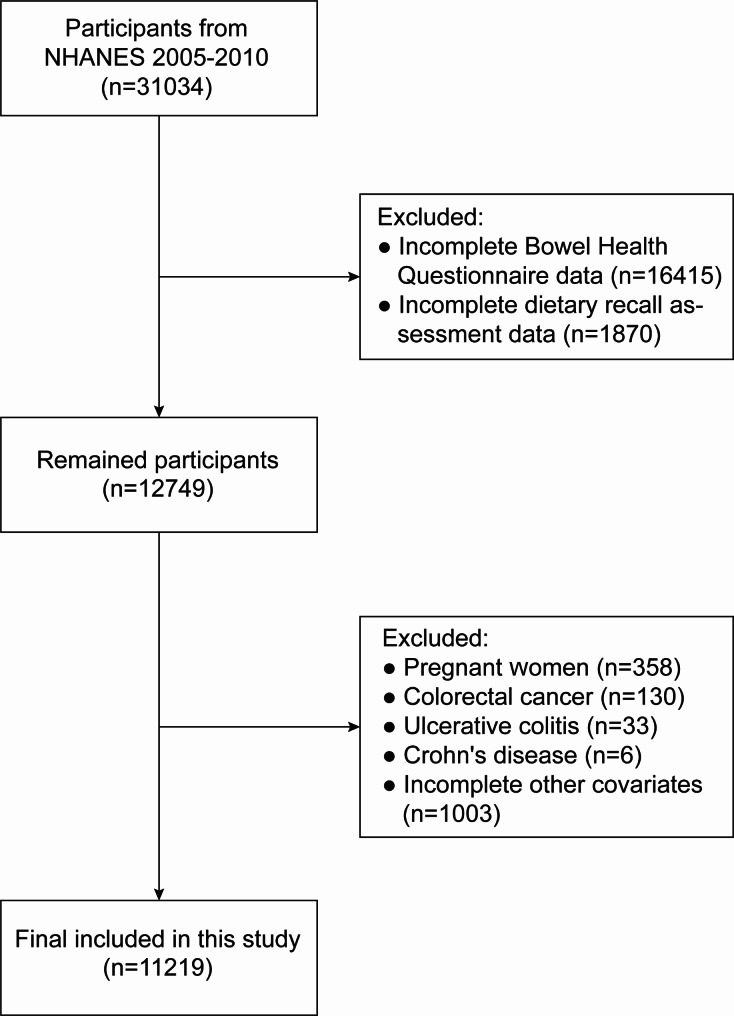
Flowchart showing how research participants were chosen from NHANES 2005–2010

The investigation used data from the Bowel Health Questionnaire (BHQ) in the survey cycles of NHANES from 2005 to 2006, 2007–2008, and 2009–2010. A total of 31,034 participants were enrolled across these cycles. After excluding 16,415 participants who lacked comprehensive BHQ information, 1,870 participants without complete dietary recall assessment data, 358 pregnant participants, 130 individuals who self-reported colorectal cancer, 33 participants with ulcerative colitis and 6 with Crohn’s disease, and an additional 1,003 participants with absent covariate data, we included 11,219 participants in our analysis (Fig. 1).

### Bowel health questionnaire

Chronic diarrhea can be assessed through a personal Bowel Health interview conducted at the Mobile Examination Center (MEC). This evaluation is identified by the variable name prefix BHQ and specifically uses question BHQ060, which employs the BSFS. Participants were asked to identify their typical or common stool type by referring to the relevant numbers on a card that featured graphic images of the seven BSFS types. Individuals who identified their typical or most frequent type of stool as either Type 1 (separate hard lumps resembling nuts) or Type 2 (sausage-like, yet lumpy) were classified as experiencing chronic constipation. Conversely, individuals who identified with Type 6 (fluffy pieces with ragged edges, a mushy stool) or Type 7 (characterized by a watery consistency, no solid pieces) were considered to be exhibiting symptoms of chronic diarrhea [24, 25].

### Dietary inflammatory index

The NHANES Nutrition Methods Workgroup collected dietary information through 24-hour recall interviews at the MEC, and we used the average nutrient intake from the two 24-hour dietary recall interviews to calculate the DII for each participant. A DII was calculated using a previously established protocol [7, 26]. Shivappa et al. [7] found that a total of 45 specific foods and nutrients were associated with various inflammatory or anti-inflammatory biomarkers, and they scored the inflammatory potential of each dietary component based on these biomarkers C-reactive protein (CRP), TNF-α, IL-1β, IL-4, IL-6, and IL-10. A score of + 1 was assigned when the dietary component significantly increased the IL-1β, IL-6, CRP and TNF-α or decreased the levels of IL-10 and IL-4. Conversely, a -1 score was allocated. Ultimately, they calculated global means and standard deviations for 45 food parameters based on 11 data sets from 11 countries. However, due to missing nutrients in the dietary database for this study, the DII was calculated based on 28 food parameters. The parameters included in the analysis were energy, carbohydrate, protein, total fatty acids, dietary fibre, total saturated fatty acids, polyunsaturated fatty acids (PUFA), monounsaturated fatty acids (MUFA), β-carotene, cholesterol, folate, niacin, iron, magnesium, selenium, zinc, alcohol, caffeine, n3 polyunsaturated fatty acids, n6 polyunsaturated fatty acids, as well as vitamins A, B1, B2, B6, B12, C, D, and E.

The calculation of the DII was performed in several steps. First, we adjusted the energy content of the nutrients using the residual method. Next, following the approach established by Shivappa et al. [7], we obtained the “global standard mean” and “global standard deviation” for each nutrient. We then calculated the z-score for each specific nutrient by subtracting the “global standard mean” from each participant’s intake and dividing this value by the “global standard deviation”. We then multiplied each participant’s z-score by the corresponding food parameter effect score [7]. Finally, we summed the DII scores for all 28 nutrients to compute the overall DII score for each participant. One’s daily dietary choices have an intrinsic pro- or anti-inflammatory potential, reflected in their overall DII. We initially analyzed DII as a continuous variable and subsequently put each participant into groups based on DII quartiles.

### Covariates

The Centers for Disease Control and Prevention (CDC) collected demographic variables, anthropometric data, self-reported health status, physical activity, alcohol consumption, and mental health information using a computer-assisted personal interview (CAPI) system. In addition, the CDC handled the processing and quality control of blood samples in the laboratory to ensure data accuracy and reliability. Covariates such as age (year), race/ethnicity, gender, marital status, education level, poverty–income ratio (PIR), body mass index (BMI, kg/m^2^), vigorous physical activity, drinking status, hypertension, diabetes, depression, serum cotinine (ng/mL), and CRP (mg/dL) were incorporated into our study as they may have a profound effect on the associations between DII and chronic diarrhea. The PIR was calculated by dividing family income by the poverty threshold, which considered household size, survey year, and region, and was then classified as < 1.3, 1.3–3.5, and ≥ 3.5. The BMI was calculated by dividing a person’s weight (in kilograms) by the square of their height (in meters), and it was categorized as 18.5–25, 25–30, and ≥ 30 kg/m^2^, corresponding to average weight, overweight and obese individuals aged 18 years or older. After carefully reviewing the information from the NHANES database physical activity questionnaire for different survey years, we found discrepancies in the definition of vigorous physical activity. The 2005–2006 cycle denoted performing any strenuous physical activity lasting at least 10 min within the past 30 days, contributing to profuse sweating or a notable increase in heart rate or breathing. In 2007–2008 and 2009–2010, participants were considered to have engaged in strenuous physical activity if it resulted in a significant increase in respiration or heart rate during work activities or recreational activities. A person was classified as a drinker if they consumed at least 12 drinks per year. Mean blood pressure was obtained from three successive measurements in a calm state. This is how hypertension was defined: (1) self-reported physician diagnosis; (2) mean diastolic blood pressure (DBP) ≥ 80 mmHg; (3) mean systolic blood pressure (SBP) ≥ 130 mmHg; and (4) taking anti-hypertensive drugs. Diabetes was identified through a self-reported history of diagnosis or glycosylated hemoglobin ≥ 6.5%. The PHQ9 questionnaire from NHANES was applied to identify patients with depression; those with scores ≥ 10 were considered to have depression [27]. The extent of smoking was expressed in serum cotinine levels. CRP was measured using latex-enhanced nephelometry.

### Statistical analysis

The statistical analyses were undertaken in accordance with CDC guidelines, incorporating NHANES sampling weights and accounting for the intricacies of multistage cluster surveys. For continuous variables, the mean ± standard deviation (SD) is utilized, whereas percentages are employed for categorical variables. We assessed differences between participants grouped by DII quartiles using weighted Student’s t-tests (continuous variables) or weighted Chi-Square tests (categorical variables). Multivariate logistic regression was used in three models to examine the independent relationship between DII and chronic diarrhea. There were no covariate modifications in Model 1. Model 2 was adjusted for gender, age, and race/ethnicity. Gender, age, race/ethnicity, education level, marital status, PIR, BMI, vigorous physical activity, drinking status, hypertension, diabetes, depression, cotinine, and CRP were all controlled for in Model 3. For the sake of examining the non-linear connections that could exist between DII and chronic diarrhea, we used Generalized Additive Models (GAMs) and smooth curve fittings. If the relationship showed non-linearity, we adopted a recursive algorithm to identify inflection points. After that, we built a two-stage linear regression model. Subgroup and interaction analyses were done for covariates, including gender, race/ethnicity, education level, BMI, PIR, vigorous physical activity, hypertension, drinking status, diabetes, and depression. Potential confounding variables were accounted for in the analysis. All statistical analyses were performed using R (version 3.4.3) and EmPower Stats (http://www.empowerstats.com). *P* values less than 0.05 were used to define statistical significance.

## Results

### Baseline characteristics

The study encompassed a sum of 11,219 participants, ranged in age from 20 to 85 years, and with an average age of 49.90 ± 17.59 years. Of them, 50.48% were women, and 49.52% were men. The DII ranged across quartiles 1–4 from − 4.94 to 0.07, 0.07 to 1.38, 1.38 to 2.48, and 2.48 to 4.69, respectively. Within the study population, 833 (7.42%) reported experiencing chronic constipation, and 836 (7.45%) reported chronic diarrhea. Among the different DII quartiles, there were notable variations in the prevalence of chronic diarrhea and chronic constipation (all *P* < 0.001). Participants across different DII quartile groups exhibited significant differences in several demographic and health-related factors, including age, gender, race/ethnicity, marital status, education level, PIR, BMI, vigorous physical activity, drinking status, hypertension, diabetes, depression, cotinine, and CRP (all *P* < 0.05). Participants in the higher DII categories had a higher likelihood of being female and having hypertension, diabetes, depression, and an abnormal BMI than those in the lowest quartile group. They engaged in less vigorous physical activity, had lower PIR, higher blood cotinine and CRP levels, and were often older (Table 1).

**Table 1:** Characteristics of participants by DII quartile in the 2005-2010 National Health and Nutrition Examination Survey (NHANES)

**Characteristics**	**Overall**	Quartiles of DII score				*p* value
		**Q1 (-4.94–0.07)**	**Q2 (0.07–1.38)**	Q3 (1.38–2.48)	Q4 (2.48–4.69)	
	N = 11,219	N = 2805	N = 2804	N = 2805	N = 2805	
Age (years, mean ± SD)	49.90 ± 17.59	50.68 ± 17.11	49.43 ± 17.02	49.21 ± 17.78	50.26 ± 18.37	0.005**
Gender						< 0.001***
Male, n (%)	5556 (49.52)	1795 (63.99)	1547 (55.17)	1243 (44.31)	971 (34.62)	
Female, n (%)	5663 (50.48)	1010 (36.01)	1257 (44.83)	1562 (55.69)	1834 (65.38)	
Race/ethnicity						< 0.001***
Mexican American, n (%)	1907 (17.00)	443 (15.79)	523 (18.65)	499 (17.79)	442 (15.76)	
Other Hispanic, n (%)	878 (7.83)	191 (6.81)	199 (7.10)	245 (8.73)	243 (8.66)	
Non-Hispanic White, n (%)	5826 (51.93)	1670 (59.54)	1487 (53.03)	1342 (47.84)	1327 (47.31)	
Non-Hispanic Black, n (%)	2184 (19.47)	381 (13.58)	488 (17.40)	618 (22.03)	697 (24.85)	
Other Races, n (%)	424 (3.78)	120 (4.28)	107 (3.82)	101 (3.60)	96 (3.42)	
Education						< 0.001***
Less than high school	2909 (25.93)	489 (17.43)	665 (23.72)	789 (28.13)	966 (34.44)	
High school	2687 (23.95)	544 (19.39)	669 (23.86)	693 (24.71)	781 (27.84)	
More than high school	5617 (50.07)	1772 (63.17)	1467 (52.32)	1321 (47.09)	1057 (37.68)	
Marital status						< 0.001***
Married	6134 (54.68)	1693 (60.36)	1615 (57.60)	1503 (53.58)	1323 (47.17)	
Widowed	906 (8.08)	183 (6.52)	215 (7.67)	202 (7.20)	306 (10.91)	
Divorced	1229 (10.95)	271 (9.66)	274 (9.77)	322 (11.48)	362 (12.91)	
Separated	339 (3.02)	65 (2.32)	91 (3.25)	85 (3.03)	98 (3.49)	
Never married	1762 (15.71)	397 (14.15)	391 (13.94)	475 (16.93)	499 (17.79)	
Living with partner	842 (7.51)	193 (6.88)	215 (7.67)	217 (7.74)	217 (7.74)	
PIR, n (%)						< 0.001***
<1.3	3177 (28.32)	546 (19.47)	737 (26.28)	847 (30.20)	1047 (37.33)	
>=1.3, < 3.5	4316 (38.47)	1002 (35.72)	1029 (36.70)	1128 (40.21)	1157 (41.25)	
>=3.5	3726 (33.21)	1257 (44.81)	1038 (37.02)	830 (29.59)	601 (21.43)	
BMI, n (%)						< 0.001***
>=18.5, < 25	2988 (26.63)	877 (31.27)	709 (25.29)	728 (25.95)	674 (24.03)	
>=25, < 30	3852 (34.33)	1023 (36.47)	1001 (35.70)	924 (32.94)	904 (32.23)	
>=30	4209 (37.52)	868 (30.94)	1060 (37.80)	1112 (39.64)	1169 (41.68)	
Vigorous physical activity, n (%)						< 0.001***
No	7437 (66.29)	1563 (55.72)	1784 (63.62)	1972 (70.30)	2118 (75.51)	
Yes	3782 (33.71)	1242 (44.28)	1020 (36.38)	833 (29.70)	687 (24.49)	
Drinking status, n (%)						< 0.001***
No	3133 (27.93)	593 (21.14)	672 (23.97)	837 (29.84)	1031 (36.76)	
Yes	8086 (72.07)	2212 (78.86)	2132 (76.03)	1968 (70.16)	1774 (63.24)	
Chronic constipation, n (%)						< 0.001***
No	10386 (92.58)	2678 (95.47)	2613 (93.19)	2586 (92.19)	2509 (89.45)	
Yes	833 (7.42)	127 (4.53)	191 (6.81)	219 (7.81)	296 (10.55)	
Chronic diarrhea, n (%)						< 0.001***
No	10383 (92.55)	2638 (94.05)	2621 (93.47)	2566 (91.48)	2558 (91.19)	
Yes	836 (7.45)	167 (5.95)	183 (6.53)	239 (8.52)	247 (8.81)	
Hypertension, n (%)						0.004**
No	5224 (46.56)	1351 (48.16)	1339 (47.75)	1307 (46.60)	1227 (43.74)	
Yes	5995 (53.44)	1454 (51.84)	1465 (52.25)	1498 (53.40)	1578 (56.26)	
Diabetes, n (%)						< 0.001***
No	9604 (85.60)	2498 (89.06)	2422 (86.38)	2352 (83.85)	2332 (83.14)	
Yes	1615 (14.40)	307 (10.94)	382 (13.62)	453 (16.15)	473 (16.86)	
Depression, n (%)						< 0.001***
No	10282 (91.65)	2674 (95.33)	2612 (93.15)	2565 (91.44)	2431 (86.67)	
Yes	937 (8.35)	131 (4.67)	192 (6.85)	240 (8.56)	374 (13.33)	
DII (mean ± SD)	1.20 ± 1.68	-1.09 ± 0.93	0.76 ± 0.37	1.94 ± 0.31	3.18 ± 0.50	< 0.001***
Cotinine (ng/mL)	60.08 ± 130.89	37.84 ± 106.00	55.54 ± 127.49	61.48 ± 131.14	86.05 ± 151.04	< 0.001***
C-reactive protein (mg/dL)	0.43 ± 0.81	0.33 ± 0.71	0.40 ± 0.61	0.46 ± 0.87	0.52 ± 0.98	< 0.001***

PIR, poverty–income ratio; BMI, body mass index; CRP, C-reactive protein. Mean ± SE for continuous variables: *P* value was calculated by weighted linear regression model. % for categorical variables: *P* value was calculated by weighted chi-square test. *P value < 0.05, **P value < 0.01, ***P value < 0.001

### Association between DII and chronic diarrhea

Table 2 presents the results of the logistic regression models, indicating a correlation between DII and chronic diarrhea. The research observed a positive correlation between higher DII scores and chronic diarrhea, which was statistically significant in both Model 1 (OR = 1.08; 95% CI, 1.04–1.13; *P* = 0.0005) and Model 2 (OR = 1.08; 95% CI, 1.03–1.13; *P* = 0.0012). However, in Model 3, a fully adjusted model, the correlation between DII and chronic diarrhea was not statistically significant (OR = 1.00; 95% CI, 0.96–1.05; *P* = 0.8501). Moreover, the DII was categorized into quartiles for analysis after being transformed from a continuous variable. In comparison to quartile 1, the multivariate-adjusted ORs for chronic diarrhea in quartiles 2, 3, and 4 were found to be 1.10 (95% CI, 0.89–1.37), 1.47 (95% CI, 1.20–1.81), and 1.53 (95% CI, 1.24–1.87), respectively, in model 1. After adjusting for race/ethnicity, gender, and age in model 2, the ORs for quartile 2, quartile 3, and quartile 4 were 1.08 (95% CI, 0.87, 1.34), 1.40 (95% CI, 1.14, 1.73), and 1.40 (95% CI, 1.14, 1.73), respectively. This implies that in Models 1 and 2, individuals in quartiles 2 through 4 showed an elevated risk of developing chronic diarrhea in comparison with those in the lowest quartile. Trend tests confirmed this relationship (*P* for trend < 0.0001). However, no similar trend was observed in Model 3, which adjusted for all confounders, with a *P* for trend of 0.3727. Additionally, gender-stratified analyses were carried out. Compared to female participants in the lowest quartile, there was a statistically significant rising trend in the risk of chronic diarrhea for female participants in the highest quartile (all *P* for trend < 0.05), which remained significant even in Model 3 (*P* for trend = 0.0192). However, among male participants, no such trend in the risk of chronic diarrhea with DII was observed (*P* for trend > 0.05).

**Table 2:** Logistic regression analysis on the association between DII and chronic diarrhea

Characteristics	Model 1 OR (95% CI)	*p* value	Model 2 OR (95% CI)	*p* value	Model 3 OR (95% CI)	*p* value
**Total** (***n* = 11,219**)						
Continuous	1.08 (1.04, 1.13)	0.0005***	1.08 (1.03, 1.13)	0.0012**	1.00 (0.96, 1.05)	0.8501
DII Quartile						
Q1	1.0		1.0		1.0	
Q2	1.10 (0.89, 1.37)	0.3754	1.08 (0.87, 1.34)	0.4867	0.96 (0.77, 1.20)	0.7189
Q3	1.47 (1.20, 1.81)	0.0002***	1.40 (1.14, 1.73)	0.0015**	1.20 (0.97, 1.49)	0.1008
Q4	1.53 (1.24, 1.87)	< 0.0001***	1.40 (1.14, 1.73)	0.0015**	1.04 (0.83, 1.30)	0.7221
*P* for trend		< 0.0001***		0.0002***		0.3727
Male (*n* = 5,556)						
Continuous	1.02 (0.95, 1.08)	0.6275	1.01 (0.94, 1.07)	0.8420	0.94 (0.88, 1.01)	0.0814
DII Quartile						
Q1	1.0		1.0		1.0	
Q2	1.14 (0.86, 1.51)	0.3776	1.14 (0.86, 1.51)	0.3729	1.02 (0.76, 1.37)	0.8880
Q3	1.45 (1.09, 1.93)	0.0097**	1.43 (1.07, 1.90)	0.0141*	1.22 (0.90, 1.64)	0.1929
Q4	0.94 (0.67, 1.32)	0.7158	0.90 (0.64, 1.28)	0.5656	0.64 (0.44, 0.93)	0.0205*
*P* for trend		0.4056		0.5472		0.2217
Female (*n* = 5,663)						
Continuous	1.15 (1.08, 1.23)	< 0.0001***	1.15 (1.08, 1.22)	< 0.0001***	1.07 (1.00, 1.15)	0.0527
DII Quartile						
Q1	1.0		1.0		1.0	
Q2	1.05 (0.75, 1.47)	0.7822	1.05 (0.75, 1.47)	0.7847	0.95 (0.67, 1.36)	0.7972
Q3	1.45 (1.06, 1.97)	0.0197*	1.45 (1.06, 1.98)	0.0199*	1.26 (0.91, 1.75)	0.1585
Q4	1.78 (1.32, 2.39)	0.0001***	1.76 (1.31, 2.37)	0.0002***	1.34 (0.97, 1.84)	0.0753
*P* for trend		< 0.0001***		< 0.0001***		0.0192*

Model 1: Non-adjusted; Model 2: Adjusted for age, gender, race/ethnicity; Model 3: Adjusted for age, gender, race/ethnicity, education level, marital status, poverty–income ratio, BMI, vigorous physical activity, drinking status, hypertension, diabetes, depression, cotinine, and C-reactive protein. *P value < 0.05, **P value < 0.01, ***P value < 0.001

**Fig. 2:**
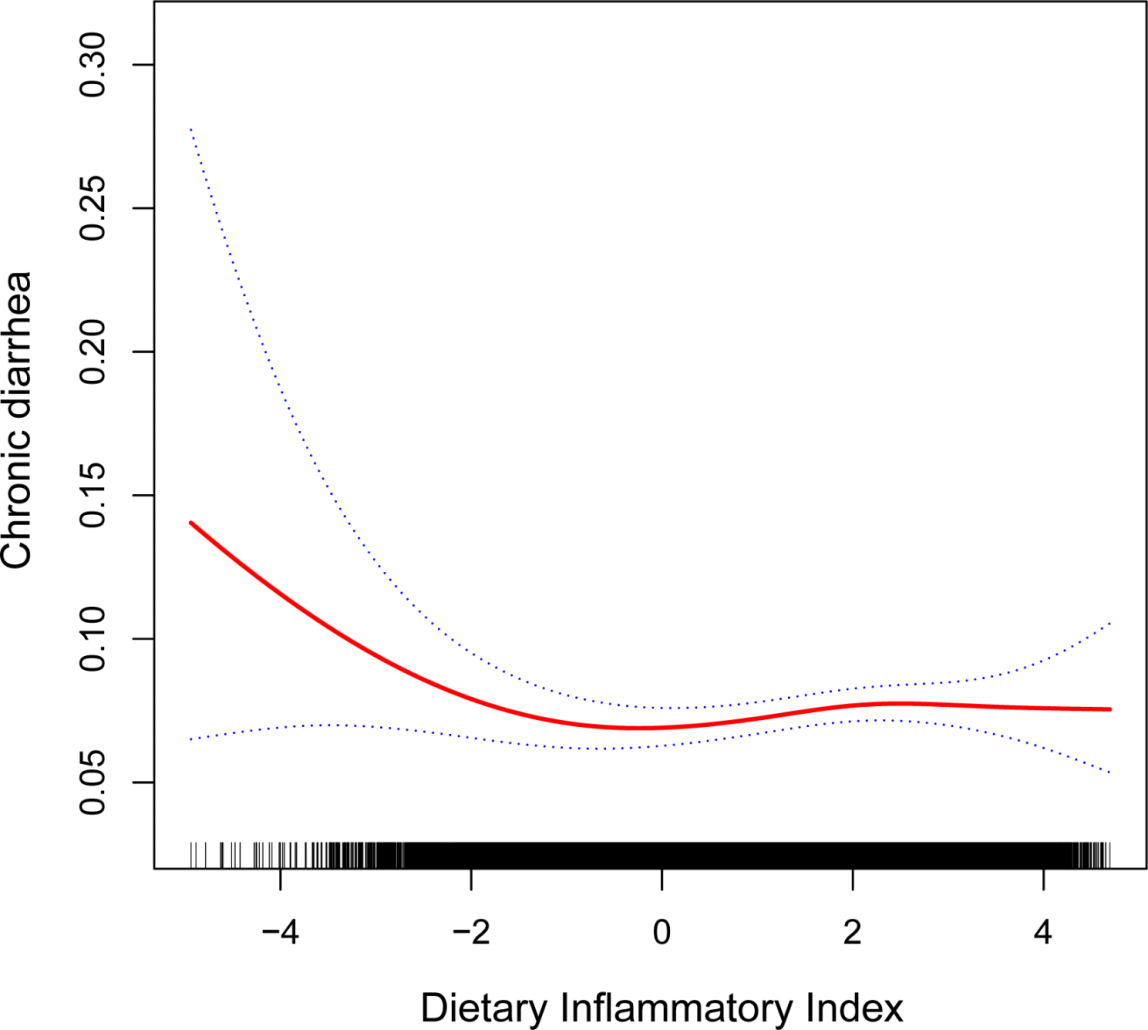
Association between DII and chronic diarrhea. Adjusted for gender, age, race/ethnicity, education level, marital status, PIR, BMI, vigorous physical activity, drinking status, hypertension, diabetes, depression, cotinine and C-reactive protein. The estimations and the associated 95% CIs are shown by the solid and dotted lines, respectively

Using GAMs and smooth curve fitting, we have uncovered an L-shaped association between DII and chronic diarrhea (Fig. 2), with the inflection point at -1.34 (Table 3). When DII values fell below - 1.34, the adjusted OR for chronic diarrhea decreased by 27% for every unit rise in DII (OR = 0.73; 95% CI, 0.57–0.93). Conversely, when DII values exceeded - 1.34, the adjusted OR for chronic diarrhea increased by 4% for every unit rises in DII (OR = 1.04; 95% CI, 0.98–1.10) (Table 3).

**Table 3:** Threshold effect analysis of DII on chronic diarrhea

	Adjust OR (95% CI)	*p* value
DII		
Fitting by standard linear model	1.00 (0.96, 1.05)	0.8501
Fitting by two-piecewise linear model		
Inflection point	-1.34	
< -1.34	0.73 (0.57, 0.93)	0.0113*
> -1.34	1.04 (0.98, 1.10)	0.1619
Log-likelihood ratio		0.014*

Adjusted for age, gender, race/ethnicity, education level, marital status, poverty–income ratio, BMI, vigorous physical activity, drinking status, hypertension, diabetes, depression, cotinine and C-reactive protein. *P value < 0.05

### Subgroup analysis

Subgroup analyses were performed, with stratification based on the following variables: gender, education level, race/ethnicity, BMI, PIR, vigorous physical activity, drinking status, hypertension, diabetes, and depression (Table 4). Only the subgroup with a normal BMI showed a statistically significant negative connection between chronic diarrhea and DII among the BMI-stratified subgroups (*P* < 0.05). Moreover, there was a positive link between the two in overweight and obese participants, but it lacked statistical significance, with ORs of 1.02 (95% CI, 0.94–1.11) and 1.05 (95% CI, 0.97–1.13), respectively (all *P* values > 0.05). No significant correlation between DII and chronic diarrhea was detected in the other subgroups (all *P* values > 0.05). The interaction between chronic diarrhea and DII demonstrated a statistically significant gender difference, according to the findings of the interaction tests (*P* interaction < 0.05).

**Table 4:** Subgroup analysis investigating the connection between DII and chronic diarrhea

Subgroup	OR (95% CI)	*p* value	*p* for interaction
Gender			0.0105*
Male	0.94 (0.88, 1.01)	0.0949	
Female	1.07 (1.00, 1.14)	0.0604	
Race/ethnicity			0.9786
Mexican American	1.02 (0.91, 1.14)	0.7449	
Other Hispanic	0.97 (0.82, 1.14)	0.7026	
Non-Hispanic White	1.02 (0.95, 1.09)	0.6125	
Non-Hispanic Black	1.00 (0.89, 1.12)	0.9472	
Other Races	0.98 (0.76, 1.26)	0.8464	
Education levels			0.1362
Less than High School	1.05 (0.96, 1.14)	0.295	
High School	1.06 (0.96, 1.17)	0.2565	
More than high school	0.95 (0.89, 1.03)	0.1961	
PIR			0.7264
< 1.3	1.03 (0.95, 1.12)	0.4755	
≥ 1.3, < 3.5	1.00 (0.93, 1.08)	0.9538	
≥ 3.5	0.98 (0.90, 1.07)	0.6761	
BMI			0.0752
>=18.5, < 25	0.90 (0.81, 0.99)	0.0311*	
>=25, < 30	1.02 (0.94, 1.11)	0.6019	
>=30	1.05 (0.97, 1.13)	0.2421	
Vigorous physical activity			0.7731
No	1.01 (0.95, 1.07)	0.7429	
Yes	0.99 (0.91, 1.09)	0.8978	
Drinking status			0.1179
No	0.98 (0.93, 1.04)	0.5063	
Yes	1.06 (0.97, 1.16)	0.1656	
Hypertension			0.322
No	0.97 (0.90, 1.05)	0.4997	
Yes	1.02 (0.96, 1.09)	0.474	
Diabetes			0.1288
No	0.99 (0.94, 1.04)	0.6184	
Yes	1.09 (0.97, 1.23)	0.1535	
Depression			0.5515
No	1.00 (0.95, 1.05)	0.9169	
Yes	1.04 (0.91, 1.20)	0.5522	

Gender, race/ethnicity, education level, marital status, PIR, BMI, vigorous physical activity, drinking status, hypertension, diabetes, and depression were all adjusted except the variable itself. *P value < 0.05

## Discussion

This cross-sectional study delved into the relationship between DII and chronic diarrhea within a U.S. population. It revealed an L-shaped relationship between DII and chronic diarrhea, indicating that DII levels were substantially linked to a heightened risk of chronic diarrhea within a specific range. These findings underscore the significance of maintaining a balanced diet that mitigates inflammation, potentially aiding in alleviating chronic diarrhea.

Chronic diarrhea can stem from various factors, including infection, abnormal immune responses, gastrointestinal protein loss, psychological factors, neuroendocrine tumors, and congenital diarrheal diseases [28]. According to a population-based study, individuals experiencing chronic diarrhea tended to have notably higher average depression scores compared to those with regular bowel habits [29]. Zhao et al. discovered a favorable relationship between depression and DII, particularly evident when DII surpassed 2.74 [30]. In our research, we found that among chronic diarrhea patients in the highest DII quartile, depression prevalence was the highest. Additionally, an elevated risk of chronic diarrhea was identified with rising DII scores, with this trend being more pronounced in female participants. Females are often more susceptible to elevated psychological stress levels and tend to adopt unhealthy dietary habits [31], which can fuel inflammation. Inflammatory-promoting diets have been connected to a heightened risk of depressive symptoms in females [32]. Hence, it is crucial to closely monitor the psychological well-being of chronic diarrhea patients, especially females.

In our fully adjusted logistic regression model, no significant association was observed between continuous DII, DII quartiles, and chronic diarrhea after accounting for all possible covariates. Through GAMs and smooth curve fitting, we identified a non-linear relationship between DII and chronic diarrhea, suggesting that the association may vary across different levels of DII, which may explain the lack of significant correlation between the two in the fully adjusted model. Additionally, as mentioned previously, higher DII scores were associated with an increased prevalence of depression, which could play a role in the relationship between DII and chronic diarrhea. Studies also showed that individuals who were overweight or obese were more prone to experience gastrointestinal dysfunction [33]. Specifically, individuals in the highest quintile of DII scores were likelier to have IBS than those in the lowest quintile [19]. These factors could significantly influence the relationship between DII and chronic diarrhea. Therefore, in our fully adjusted Model 3, the control for these covariates further elucidates the lack of a notable association between DII and chronic diarrhea.

Another study discovered that individuals in the highest DII quartile were more likely than people in the lowest quartile to experience chronic diarrhea [34]. It also revealed a positive correlation between dietary inflammation levels and abnormal gut health [34]. Those favoring a pro-inflammatory diet were more prone to gastrointestinal distress than those adhering to an anti-inflammatory diet [35]. Increased intestinal inflammation might create a conducive environment for intestinal pathogens, rendering individuals more susceptible to bacterial gastrointestinal infections, which could lead to diarrhea [35]. The “food hypothesis” provides us with the basis. Pro-inflammatory diets and intestinal inflammation can alter the nutritional spectrum, which may lead to the disruption of the mucosal barrier by pathogenic intestinal bacteria and parthenogenetic intestinal pathogens [36]. During inflammatory states, the intestinal mucosa releases antimicrobial effector mechanisms that may selectively inhibit or kill much of the intrinsic microbiota. This can further exacerbate the inflammatory state and trigger diarrheal symptoms [36].

A pro-inflammatory diet can elicit chronic and sustained immune system activation, resulting in mild inflammation [37]. For instance, the Western diet (WD), characterized by high dietary fat and low fiber, vitamins, and minerals, has been implicated in promoting inflammation and affecting metabolic and immune system functions [38]. Research involving both mice and human subjects has revealed elevated levels of inflammatory markers in their serum when exposed to the WD [39, 40], indicating a potential direct or indirect immune system response to this dietary pattern. In contrast, the Mediterranean diet, rich in dietary fiber and antioxidant foods, has been shown to reduce inflammation and enhance endothelial function [41]. Experiments in mice have shown that supplementation with vitamin D or A, fiber, or indole can ameliorate intestinal inflammation by regulating the CD4 + T cell phenotype and restoring the production of short-chain fatty acids (SCFAs) [42].

An inflammatory diet may diminish the number of beneficial microorganisms that safeguard the gut barrier, such as Bifidobacterium spp, Lactobacillus spp, Bacteriodetes spp, and Clostridiales spp, while simultaneously increasing those that compromise gut barrier integrity [43–45]. The integrity and dynamic equilibrium of the intestinal barrier depend heavily on tight junction (TJ) proteins. Consuming a diet high in pro-inflammatory compounds has been demonstrated to reduce TJ protein expression in mice, resulting in heightened intestinal permeability [46]. In contrast, piglets weaned and fed bran fiber experienced an upregulation in the expression of the TJ protein zonula occludens (ZO)-1 in the intestine, leading to a reduced incidence of diarrhea [47]. Wu et al. have demonstrated that pro-inflammatory diets can instigate colonic inflammation by disrupting the metabolic balance of amino acids, bile acids, and fatty acids, consequently affecting inflammatory gene expression [48]. Individuals with the diarrhea subtype of IBS were found to have more healthy plant foods, magnesium, and iron, but they exhibited reduced gut microbial diversity and fewer butyrate-producing anaerobic bacteria [5]. SCFAs, including butyrate, serve as essential fuels for intestinal epithelial cells (IECs), influencing intestinal motility and enhancing intestinal barrier function by regulating IEC proliferation and differentiation [49]. Furthermore, SCFAs exert anti-inflammatory effects by modulating immune cell function and cytokine production [50]. Butyrate salts can inhibit the expression of inflammatory factors such as MCP-1, IL-6, TNF-α and by activating macrophage GPR41 [51]. Therefore, a pro-inflammatory diet disrupts intestinal homeostasis by inducing intestinal microbiota dysbiosis and damaging the intestinal mucosal barrier. These alterations can elevate the risk of diarrhea and even lead to intestinal inflammation.

This study’s primary strengths include its use of a large, nationally representative NHANES dataset, offering valuable insights into dietary factors and health outcomes across the U.S. population, and its control of confounders such as comorbidities and depression, enhancing the findings’ reliability. Our findings suggest that dietary interventions could effectively manage chronic diarrhea, particularly for individuals following a pro-inflammatory diet. Clinicians may improve patient management and guide nutritional adjustments by assessing and modifying dietary inflammation levels using the DII. Public health initiatives targeting pro-inflammatory diets could offer preventive support by educating the public on inflammatory dietary components, promoting healthier choices, and potentially reducing the burden of gastrointestinal issues linked to inflammatory diets.

However, several limitations must be considered. First of all, its cross-sectional design prohibits determining whether the impact of DII on chronic diarrhea varies over time or allows for assessing causality. Secondly, relying on self-reported 24-hour dietary recalls and the BSFS to evaluate DII and chronic diarrhea heightens the risk of recall bias. Thirdly, although a range of covariates was included to reduce confounding bias, unknown or unmeasured confounders may still affect the results, given the complex aetiology of chronic diarrhea. Finally, while our study population comprised individuals experiencing diarrhea, we could not entirely exclude participants with IBS, which complicates investigating the relationship between pro-inflammatory diets and different types of diarrhea. Future research should focus on addressing these limitations.

## Conclusion

The study reveals a correlation between elevated DII levels and an increased risk of chronic diarrhea. These findings underscore the significance of dietary inflammation in identifying individuals susceptible to chronic diarrhea. However, it is crucial to conduct further large prospective studies to validate and further elucidate these findings.

## Data Availability

The public may easily view the related data in this study at https://www.cdc.gov/nchs/nhanes/.
